# Analgesic effects and metabolome analyses of laser- and electro-acupuncture combined therapies in paclitaxel-induced neuropathic pain model

**DOI:** 10.3389/fvets.2023.1153903

**Published:** 2023-04-18

**Authors:** Chan-Suk Yoon, Ga-Won Lee, Myeong-Hun Kim, Sang-Mi Kang, Cha-Kyung Youn, Ji-Hye Yang, Eun-Ju Kim, Hong-Seok Son, Sok Cheon Pak, Seon-Jong Kim, Chang-Su Na

**Affiliations:** ^1^School of Korean Medicine, Dongshin University, Naju, Jeonnam, Republic of Korea; ^2^Department of Companion Animal Industry, College of Health and Welfare, Dongshin University, Naju, Jeonnam, Republic of Korea; ^3^Department of Biotechnology, College of Life Sciences and Biotechnology, Korea University, Seoul, Republic of Korea; ^4^School of Biomedical Sciences, Charles Sturt University, Bathurst, NSW, Australia

**Keywords:** laser-and electro-acupuncture combined therapy, paclitaxel, allodynia, endocannabinoids, metabolome analyses

## Abstract

**Introduction:**

Allodynia, which can be induced by paclitaxel administration, is the presence of pain as a result of a stimulus that does not usually provoke pain. Many studies have investigated the analgesic efficacy of acupuncture, including laser acupuncture (LA) and electroacupuncture (EA). Although pain-related diseases are relatively common, few studies have analyzed the analgesic effects and mechanisms of LA combined with EA. The purpose of this study was to investigate the therapeutic effect and mechanism of manual acupuncture (MA), EA, LA, and combined therapy (LA + EA) in a paclitaxel-induced allodynia rat model.

**Methods:**

A total of 56 rats were classified into eight groups: a normal (Nor, *n* = 7), a control (Con, *n* = 7), an MA (*n* = 7), an EA (*n* = 7), a 650-nm LA (650LA, *n* = 7), an 830-nm LA (830LA, *n* = 7), a 650-nm LA combined with EA (650LA + EA, *n* = 7), and an 830-nm LA combined with EA group (830LA + EA, *n* = 7). Allodynia was induced by intraperitoneal injection of 2 mg/kg of paclitaxel every other day for a total of four times except the Nor group. Acupuncture treatments were conducted at the points of Jungwan (CV12) and Joksamni (ST36) once every other day for 6 min, for a total of nine times. Withdrawal response reaction times and force intensity of the foot were measured before the start of the experiment, after the 4th paclitaxel administration (day 8), and after the 9th and last treatment (day 15). On the 16th day, mRNA and protein expression in the spinal nerves was assessed, and a metabolome analysis of the animals’ feces was performed.

**Results and discussion:**

Our analyses show that 650LA + EA treatment resulted in an upregulation of protein expression related to pain relief and nerve regeneration, whereas 830LA + EA treatment led to significant changes in metabolomes. This study demonstrates that a combination treatment of EA and LA can suppress allodynia and promote upregulation of protein expression related to nerve regeneration and is effective in changing the intestinal microbiome. Further large-scale research is required to assess the exact mechanism underlying the therapeutic effect of this combination treatment in pain-related diseases.

## Introduction

The expression of pathological pain experienced by patients in clinical practice varies greatly between individuals. Complaints can be divided into those of spontaneous pain, hyperalgesia, and allodynia, which is defined as pain caused by stimuli that do not normally cause pain (1). Allodynia, that is, abnormal stimulus-induced nociceptive pain, can be classified into dynamic mechanical, static mechanical, heat, and motor allodynia (2, 3). Otherwise, hyperalgesia is an increased pain due to a stimulus which usually provokes pain (4). Neuropathic pain, which is spontaneous and persistent, often requires adjuvant treatment, such as combined analgesic administration or other interventional pain treatments (2, 5). Propagation of nociceptive pain is transmitted to the somatosensory cortex by secretion of glutamate, γ-aminobutyric acid (GABA), or substance-P from the presynaptic neurons of peripheral nerves and binding to postsynaptic neurons (6). Neuronal pain processing, perception, and modulation have very complex mechanisms, and various proteins including cannabinoid receptor type 1 (CB1R) and fatty acid amide hydrolase (FAAH), c-Fos proto-oncogene (c-Fos), neuronal nuclear antigen (NeuN), and neuronal phosphoproteins, including synapsin I and microtubule-associated protein 2 (MAP2) are involved in the pain pathways (7). CB1R and FAAH belong to endocannabinoid system, which interacts with various endogenous ligands and neurotransmitters with neuroprotective, analgesic, and anti-inflammatory properties (8). c-Fos is present in neurons in response to stimulation, and synapsin 1 regulates synapse formation and neurotransmitter release (9, 10). NeuN and MAP2 regulate neuronal growth and regeneration (11–13). These are closely related to analgesic effects by the inhibition of presynaptic neurotransmitter and neuropeptide release, modulation of postsynaptic neuron, and reductions in neuroinflammatory signaling (14).

In clinical practice, paclitaxel is used alone or in combination for the treatment of breast, lung, ovarian, and gastric cancer; it inhibits the differentiation and cell cycle arrest in the Sub-G1 and G2/M phases, eventually leading to apoptosis (15). Cell surface changes, cell destruction, and formation of apoptotic bodies by phagocytes occur, which then proceed as a result of the action of the immune system (16). However, paclitaxel inhibits tumor growth and affects normal cells simultaneously, resulting in damage to mucous membranes, blood vessels, and neurons with a short cell cycle; this causes neuropathic side effects such as pain, tingling, cold sensitivity, and numbness (17). These side effects are thought to be caused by an immune response following the production of pro-inflammatory mediators and the development of central sensitization and pain behavior caused by paclitaxel administration (18).

Many studies have investigated the efficacy of acupuncture in Oriental medicine. Acupuncture contributes to angiogenesis and granulation and promotes tissue regeneration of wounds in mice (19). Other studies have reported adjustment of the primary somatosensory cortex by acupuncture (20), improvement of colitis by electroacupuncture (EA) treatment of sensitive skin points and acupuncture points (21), and an insomnia treatment effect of acupuncture (22). In addition, the combination of EA and laser acupuncture (LA) with modern science and technology as well as with traditional acupuncture is used as an acupoint treatment method. Previous studies have reported combined effects of LA and EA in a collagenase-induced osteoarthritis rat model (23) and in human patients with knee osteoarthritis (24).

Among the various acupoints, Jungwan (CV12) and Joksamni (ST36) can easily be designated in experimental animals through bone proportional measurements (25). ST36 acupuncture is used for pain relief and immunity, such as suppression of IgE and the effect of Th1/Th2 regulation (26), while CV12 is the mother blood of the stomach, which is a specific acupuncture point where the energy of the intestines gathers (27). These two acupoints are assigned to meridians that are closely associated with the spleen and stomach and are related to intestinal microorganisms and the brain–gut microbiota axis, which is involved in the body’s immunity and control of the brain (28).

The objective of this study was to evaluate the analgesic effect and mechanism of manual acupuncture (MA), EA, LA, and the combination therapy of LA and EA by assessing pathological pain through von Frey filaments and analyses of mRNA and protein expression as well as metabolomes in a paclitaxel-induced allodynia rat model.

## Materials and methods

### Animals and study design

A total of 56 10-week-old Sprague Dawley rats weighing approximately 330–350 g, were included in this study. They were first allowed to adapt to the laboratory environment, with a sufficient supply of feed and water and at a constant temperature of 24 ± 1°C and humidity of 40–60% for 7 days. Water and feed were provided *ad libitum* during the entire experimental period. The study was approved by the Committee of Animal Care and Experiments of Dongshin University, Republic of Korea (DSU2020-04-01). The rats were randomly divided into eight groups: a normal group (Nor, *n* = 7), a control group (Con, *n* = 7), a MA group (*n* = 7), an EA group (*n* = 7), a 650-nm LA group (650LA, *n* = 7), an 830-nm LA group (830LA, *n* = 7), a 650-nm LA combined with EA group (650LA + EA, *n* = 7), and an 830-nm LA combined with EA group (830LA + EA, *n* = 7; Table 1). Except in the Nor group, 2 mg/kg paclitaxel was injected intraperitoneally every 2 days for a total of four times to induce allodynia.

**Table 1:** Experimental group classification of acupuncture treatments for the paclitaxel-induced allodynia model.

Group	Acupoint	Contents
Nor	–	–
Con	–	PT (2 mg/kg, IP)
MA	CV12, ST36	PT (2 mg/kg, IP) + MA
EA	CV12, ST36	PT (2 mg/kg, IP) + EA
650LA	CV12, ST36	PT (2 mg/kg, IP) + 650 nm LA
830LA	CV12, ST36	PT (2 mg/kg, IP) + 830 nm LA
650LA + EA	CV12, ST36	PT (2 mg/kg, IP) + 650 nm LA + EA
830LA + EA	CV12, ST36	PT (2 mg/kg, IP) + 830 nm LA + EA

IP, intraperitoneal injection; PT, paclitaxel; MA, manual acupuncture; EA, electro acupuncture; LA, laser acupuncture; Nor, normal; Con, control.

**Figure 1:**
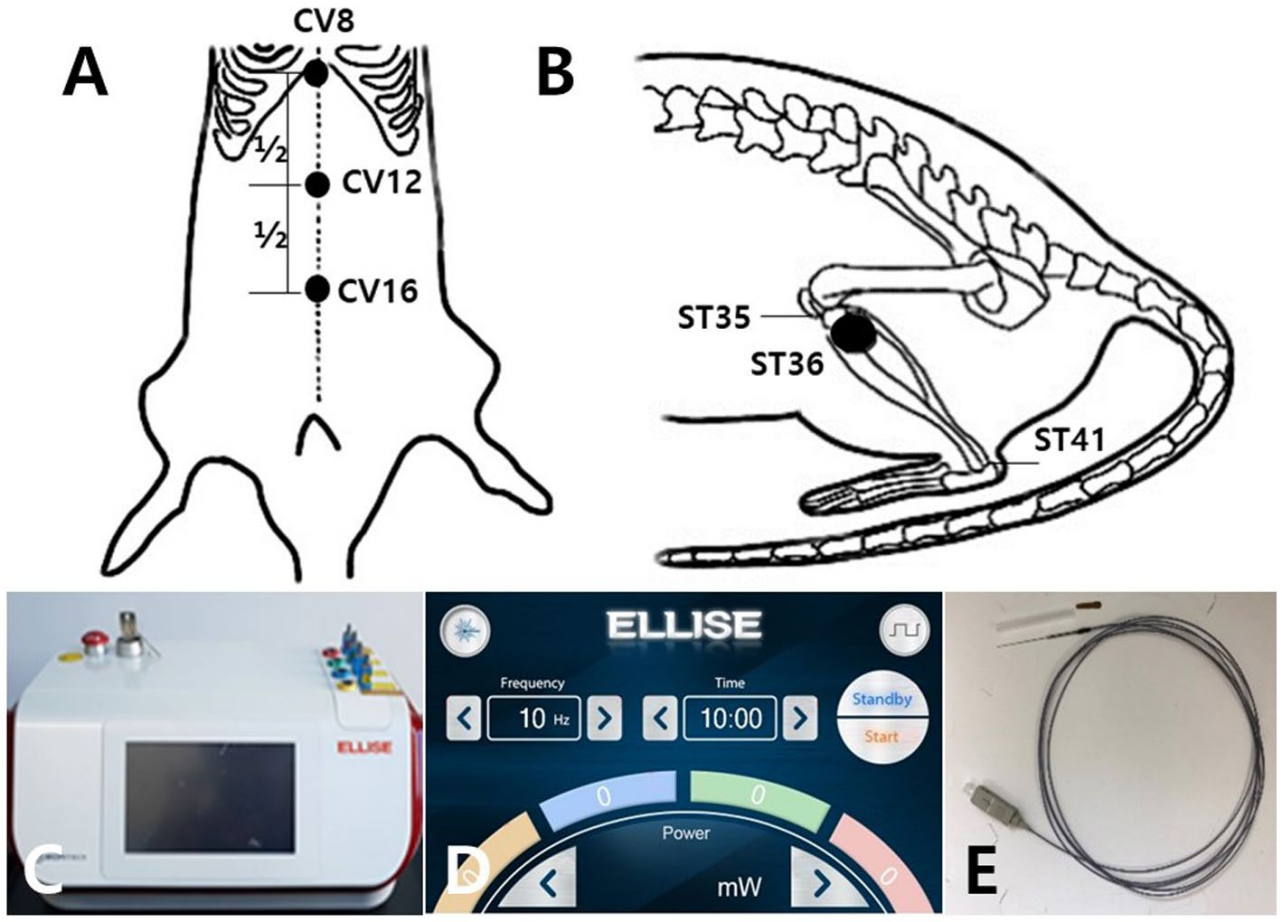
Schematic diagram of acupoints CV12 and ST36 and the laser and electrical stimulator System. Schematics show the acupuncture points CV12 **(A)** and ST36 **(B)**. CV12 was located at the median point of the abdominal midline connecting the umbilicus and sternum **(A)**. ST36 was located in the tibialis anterior muscle **(B)**. The laser and electrical stimulator system (ELLEISE, WONTECH Co., Ltd., Republic of Korea) used for laser acupuncture and electrical stimulation is shown, including the main body of the system **(C)**, the laser output adjustment screen **(D)**, and the fiber-optic acupuncture cable **(E)**.

All acupuncture treatments were performed at CV12 and ST36 once every other day for 6 min, for a total of nine times. EA was conducted at 50 Hz and 4 mA and LA at 50 Hz and 20 mW. The acupuncture points were CV12 on the upper abdomen (Figure 1A), and ST36 on the anterior aspect of the leg (Figure 1B). The anatomical locations of the acupoints were stimulated as previously described (29, 30). EA needles with a diameter of 0.3 mm were inserted into the skin and underlying muscles with a depth of 3–4 mm. In the case of the ST36 position, the right and left side was stimulated alternately at each treatment cycle. For the LA and EA combination treatment, 3 min of LA treatment were followed by 3 min of EA treatment. Table 2 shows the specific conditions of the stimulator device used for the combined application of laser and electric acupuncture. A laser and electrical stimulator system (ELLEISE; WONTECH Co. Ltd., Republic of Korea) was used for laser acupuncture and electrical stimulation, and an optical fiber was inserted into an empty stainless cannula for invasive laser acupuncture stimulation (Figures 1C–E). During all acupuncture treatments including EA and LA, rats were under general anesthesia by inhalation anesthetics with 2.5% isoflurane and 80% O_2_.

**Table 2:** Specifications of the laser and electrical stimulator system.

No	Classification		Specification
1	Wavelength		650 nm ± 20 nm, 830 nm ± 20 nm
2	Laser diode		InGaAlP (650 nm), GaAlAs (830 nm)
3	Laser transmission method		Fiber-optic acupuncture
4	Laser irradiation diameter		0.15 mm ± 20%
5	Laser Output	Optical fiber beam transmission device (optical fiber)	650 nm: 10 mW, 20 mm, 30 mm ± 20% 830 nm: 10 mm, 20 mm, 30 mm ± 20%
		Modulation frequency	650 nm: 1 ~ 200 Hz 830 nm: 1 ~ 200 Hz
6	Electrical stimulator	Voltage	Max. 5.0 V/0.1 V increase or decrease in units
		Output waveform	Sine, Triangular, Pulse
		Modulation frequency	1 Hz ~ 200 Hz
7	Output time		Min 1 min ~ Max 30 min/1 min unit
8	Type and degree of protection against electric shocks		Grade 1, BF type
9	Laser classification by IEC 60825–1		CLASS 3B
10	User interface		Touch LCD method (7 inches)
11	Equipment size		400 mm (W) × 259 mm (D) × 200 mm (H)
12	Weight		4.58 kg
13	Cooling system		Air-cooled
14	Electrical rating		AC 220 V, 60 Hz Power consumption: 200VA

Withdrawal response reaction times and force intensity in the foot were measured using a dynamic plantar aesthesiometer three times: before the start of the experiment, after the 4th paclitaxel administration (day 8), and after the 9th and last treatment (day 15). On the 16th day, all animals were euthanized, sections of lumber (L3–L6) and sacral (S1, S2) segments of the spinal cord and feces were collected, RNA was isolated from the spinal nerves, and a metabolome analysis of the feces was performed. The mRNA expression of CB1R and FAAH and the protein expression of c-Fos, CB1R, FAAH, NeuN, synapsin I, and MAP2 were analyzed.

### Pain behavioral test

Withdrawal response reaction times and force intensity were measured by stimulating the sole of the foot with von Frey stimuli using a dynamic plantar aesthesiometer (UGO BASILE 37450, Italy) three times: before the start of the experiment, after the 4th paclitaxel administration (day 8), and after the 9th and last treatment (day 15). For the measurement, the animal was moved to a cage made of netting and stabilized for 5 min; then the withdrawal response was assessed. The degree of allodynia caused by the stimulation was measured as the withdrawal response reaction time of the foot (in seconds; time taken for the stimulator to touch the sole of the foot and fall off) and the force intensity (in g, force at the moment the animal avoids the stimulator) on the affected side at an intensity of 0–50 g.

### RNA isolation and reverse transcription-polymerization chain reaction (RT-PCR)

For isolation of RNA, 1 ml TRIzol Reagent (Life Technologies, United States) were added to spinal nerve tissue (100 mg) and homogenized in a homogenizer (Precellys 24, Bertin Technologies, France), with 200 μl chloroform (Sigma, United States) added to the homogenate. After shaking for 15 s, the mixture was left at room temperature for 5 min and then centrifuged for 5 min at 4°C and 14,000 rpm in a centrifuge (Centrifuge 5415 R; Eppendorf, Germany) to remove cell residues. Then, the supernatant was separated, 500 μl isopropanol (Sigma, United States) were added, the mixture was left at room temperature for 5 min, and centrifuged again at 4°C and 14,000 rpm for 8 min. Diethylpyrocarbonate was added to the obtained RNA pellet in 70% ethanol and stored in a refrigerator. The pellet was separated by centrifugation at 4°C and 7,500 rpm for 5 min, and the remaining ethanol was left at room temperature for 5 min to evaporate, then dissolved in diethylpyrocarbonate-treated water. RNA purity and concentration were measured using the OD260 value on a spectrophotometer (Biophotometer, Eppendorf, Germany). A cDNA Synthesis Master Mix (LeGene Biosciences, United States) was used for amplification by a cDNA synthesis process at 42°C for 1 h and an RTase inactivation process at 94°C for 5 min, on a Mastercycler gradient machine (Eppendorf, Germany).

SB-Green qPCR Master Mix (LeGene Biosciences, United States) was used for amplification on a CFX Connect Optics Module (BIO RAD, Singapore). PCR included a 2-min pre-denaturation process at 95°C and a 50-s denaturation process at 95°C, and the Tm (°C) of each gene was a 10-s annealing process and a 30-s extension process at 72°C. A repeat process of 39 cycles was performed, and cDNA was amplified through a melting process.

### Western blot

The tissue was homogenized using pro-prep protein extraction buffer and beads, then incubated on ice for 30 min and centrifuged at 13,300 rpm for 20 min at 4°C; the supernatant was collected. After quantifying the protein following the BCA method, 20 μg of the corresponding amount was put into 5 × sample buffer, inactivated at 100°C for 5 min, and then electrophoresed on a 10% SDS polyacrylamide gel. The separated proteins were transferred to a PVDF membrane and blocked in 5% skim milk for 60 min. The primary antibody was incubated overnight at 4°C, washed three times for 5 min with Tris-buffered saline Tween-20 (TBST, 10 mM Tris–HCl, 150 mM NaCl, 0.05% Tween 20, pH 7.6) buffer, and the secondary antibody was incubated at 1:5,000 and attached at room temperature for 2 h. For protein detection, the membrane was washed with TBST buffer three times for 10 min, ECL (Thermo, United States) was applied to the membrane for 2 min, and measurements were performed using LAS equipment (Amersham imager 600; GE, United States).

### Metabolome analysis

Hundred uL of O-methoxyamine hydrochloride/pyridine (15 mg/ml) were added to the lyophilized fecal sample, vortexed for 1 min, ultrasonicated for 10 min, and incubated in the dark (90 min, 30°C, 75 rpm). After the reaction was completed, 50 μl of N-methyl-N-(trimethlysilyl)trifluoroacetamide with 1% trimethylchlorosilane were added for vortexing (1 min), and the solution was left to react at 37°C for 30 min, followed by vortexing for 2 min and centrifugation at 13,000 rpm for 10 min; 100 uL of the supernatant were then placed in a vial, and gas chromatography–mass spectrometry (GC–MS) analysis was performed.

The metabolome analysis of the derivatized samples was performed by GC–MS using a QP2020 GC–MS instrument (Shimadzu; Kyoto, Japan). Rtx-5MS (30 μL × 0.25 mm × 0.25 μM) was used as the analysis column, and the GC oven temperature was maintained at 80°C for 2 min, then raised to 330°C at 15°C/min and maintained again for 5 min. The temperatures of the MS interface and filament source were set to 250 and 200°C, respectively, and the scan range was set to 85–500 m/z.

After changing the raw data file for GC–MS pre-processing to a netCDF file, peak detection and alignment were performed using Metalign software. The files were then subjected to peak identification using AIoutput software. The pre-processed data were subjected to principal component analysis (PCA), partial least squares-discriminant analysis (PLS-DA), and permutation tests using SIMCA software (SIMCA 15.0; Umetics, Sweden). Metabolome identification was performed after comparison with the mass spectra, NIST 14.0 library, and HMDB.

### Statistical analysis

Statistical analyses were performed using GraphPad Prism Statistics software version 6.01 (United States). All continuous data are presented as means ± standard error. Normal distribution assumption was confirmed using the Kolmogorov–Smirnov test. Values were compared between groups using one-way ANOVA and Tukey’s multiple comparisons test. A *p* value of < 0.05 was considered statistically significant. The PLS-DA score was analyzed by the permutation test analysis using SIMCA software (SIMCA 15.0; Umetics, Sweden).

## Results

### Withdrawal response reaction times and force intensity

Table 3 shows the effect of each treatment on the von Frey reaction times for paclitaxel-induced allodynia. Compared to the Nor group, the Con group showed significantly shorter von Frey reaction times on days 8 and 15 (*p <* 0.01). Compared to the Con group, the MA, EA, and 650LA + EA groups showed significantly longer von Frey reaction times on day 8 (*p <* 0.01), and the EA and 830LA + EA groups showed significantly longer reaction times on day 15 (*p <* 0.01).

**Table 3:** Changes in von Frey reaction times in rats with paclitaxel-induced allodynia according to type of acupuncture treatment.

Group	Day 0 (sec)	Day 8 (sec)	Day 15 (sec)
Nor	6.42 ± 0.23	6.32 ± 0.29	6.40 ± 0.16
Con	6.62 ± 0.62	2.82 ± 0.31^##^	3.53 ± 0.56^##^
MA	6.43 ± 0.38	4.78 ± 0.35^**^	5.32 ± 0.51
EA	6.70 ± 0.52	4.93 ± 0.49^**^	5.68 ± 0.26^**^
650LA	6.57 ± 0.40	4.08 ± 0.53	4.83 ± 0.19
830LA	6.47 ± 0.45	3.78 ± 0.67	5.38 ± 0.56
650LA + EA	6.62 ± 0.21	4.70 ± 0.17^**^	5.42 ± 0.60
830LA + EA	6.37 ± 0.49	3.87 ± 0.62	6.23 ± 0.24^**^

^##^*p* < 0.01 compared with the normal group; *p < 0.05, **p < 0.01, compared with the control group.

MA, manual acupuncture; EA, electro acupuncture; LA, laser acupuncture; Nor, normal; Con, control; sec, seconds.

Table 4 shows the effect of each treatment on the von Frey force intensity for paclitaxel-induced allodynia. Compared to the Nor group, von Frey force intensity in the Con group was significantly lower on days 8 and 15 (*p <* 0.01). Compared to the Con group, significantly higher von Frey force intensity was observed in the MA, EA, and 650LA + EA groups on day 8 (*p <* 0.05), and in the EA and 830LA + EA groups on day 15 (*p <* 0.01).

**Table 4:** Changes in von Frey force intensity in the foot of rats with paclitaxel-induced allodynia according to type of acupuncture treatment.

Group	Day 0 (g)	Day 8 (g)	Day 15 (g)
Nor	35.75 ± 1.25	35.23 ± 1.59	35.69 ± 0.90
Con	36.88 ± 3.42	18.43 ± 1.17^##^	20.82 ± 3.60^##^
MA	35.85 ± 2.11	26.78 ± 1.95^**^	28.97 ± 2.49
EA	37.32 ± 2.86	27.60 ± 2.73^*^	31.72 ± 1.45^*^
650LA	36.58 ± 2.17	22.93 ± 2.93	27.05 ± 1.04
830LA	36.05 ± 2.47	21.30 ± 3.70	34.02 ± 3.34^*^
650LA + EA	36.87 ± 1.14	26.33 ± 0.93^**^	35.65 ± 2.68^*^
830LA + EA	35.47 ± 2.69	22.02 ± 3.42	34.75 ± 1.33^**^

^##^*p* < 0.01 compared with the normal group; *p < 0.05, **p < 0.01, compared with the control group.

MA, manual acupuncture; EA, electro acupuncture; LA, laser acupuncture; Nor, normal; Con, control; g, grams.

### mRNA expression of CB1R and FAAH

**Figure 2:**
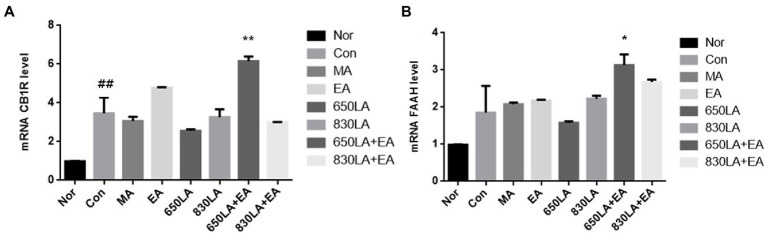
mRNA expression of CB1R and FAAH in rats with paclitaxel-induced allodynia according to type of acupuncture treatment. There was a significant increase in mRNA CB1R in the Con group compared to the Nor group (*p <* 0.01), and a significant increase in mRNA CB1R in the 630LA + EA group compared to the control group (*p <* 0.01, **A**). Compared to the Nor group, the Con group had a tendency to an increase in FAAH mRNA expression and a significant increase in mRNA FAAH in the 630LA + EA group compared to the control group (*p <* 0.05, **B**). ^##^*p <* 0.01 compared to the normal group; ^*^p < 0.05, compared to the control group; ^**^p < 0.01, compared to the control group. MA, manual acupuncture; EA, electroacupuncture; LA, laser acupuncture; Nor, normal; Con, control.

CB1R mRNA expression in the Nor, Con, MA, EA, 650LA, 830LA, 650LA + EA, and 830LA + EA group was 0.98 ± 0.01, 3.47 ± 0.23, 3.06 ± 0.18, 4.77 ± 0.03, 2.56 ± 0.07, 3.60 ± 0.18, 6.15 ± 0.19, and 2.99 ± 0.02, respectively (Figure 2A). There was a significant increase in CB1R mRNA in the Con group compared to the Nor group (*p <* 0.01), and a significant increase in CB1R mRNA in the 630LA + EA group compared to the Con group (*p <* 0.01).

The mRNA expression of FAAH in the Nor, Con, MA, EA, 650LA, 830LA, 650LA + EA, and 830LA + EA group was 0.98 ± 0.01, 1.85 ± 0.48, 2.08 ± 0.04, 2.17 ± 0.02, 1.58 ± 0.04, 2.22 ± 0.07, 3.13 ± 0.24, and 2.68 ± 0.05, respectively (Figure 2B). Compared to the Nor group, the Con group showed a tendency to increased FAAH mRNA levels, while the 630LA + EA group showed a significant increase in FAAH mRNA compared to the Con group (*p <* 0.05).

### Protein expression of c-Fos, CB1R, FAAH, NeuN, synapsin I, and MAP2

**Figure 3:**
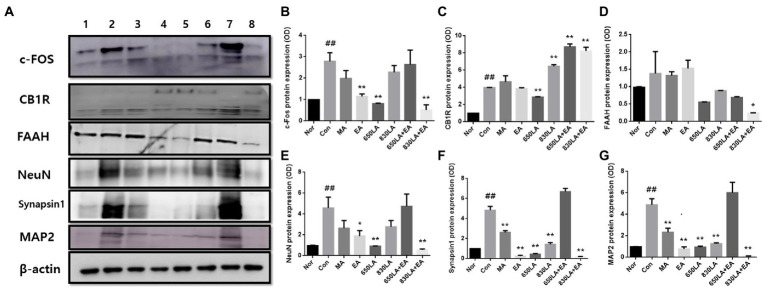
Protein expression in rats with paclitaxel-induced allodynia according to type of acupuncture treatment. The protein expression of c-Fos, CB1R, FAAH, NeuN, synapsin I, and MAP2 is shown **(A)**. The EA, 650 LA, and 830 LA + EA groups had significantly decreased c-Fos protein levels compared to the Con group (*p <* 0.01, **B**). There was a significant decrease in CB1R protein expression in the 650LA group and a significant increase in CB1R protein expression in the 830LA, 650LA + EA, and 830LA + EA groups compared to the Con group (*p <* 0.01, **C**). FAAH protein levels were significantly lower in the 830LA + EA group than in the Con group (*p <* 0.05, **D**). The EA, 650LA, and 830LA + EA groups had significantly lower NeuN protein levels than the Con group (*p <* 0.05, *p <* 0.01, and *p <* 0.01, respectively, **E**). The MA, EA, 650LA, 830LA, and 830LA + EA groups had significantly lower synapsin I protein levels than the Con group (*p <* 0.01, **F**). The MA, EA, 650LA, 830LA, and 830LA + EA groups had significantly lower MAP2 protein levels than the Con group (*p <* 0.01, **G**). Only the 650 LA + EA group showed a tendency to increase NeuN, synapsin I, and MAP2 compared to the control group, although the difference was not significant. 1, Nor; 2, Con; 3, MA; 4, EA; 5, 650LA; 6, 830LA; 7, 650LA + EA; 8, 830LA + EA. ^##^*p <* 0.01 compared to the normal group; ^*^p < 0.05, compared to the control group; ^**^p < 0.01, compared to the control group. MA, manual acupuncture; EA, electroacupuncture; LA, laser acupuncture; Nor, normal; Con, control.

Figure 3A shows Western blotting results revealing the effect of each treatment on the expression of pain-related proteins, including c-Fos, CB1R, FAAH, NeuN, synapsin I, and MAP2.

The c-Fos protein expression in the Nor, Con, MA, EA, 650LA, 830LA, 650LA + EA, and 830LA + EA group was 0.98 ± 0.01, 2.79 ± 0.33, 1.98 ± 0.30, 1.15 ± 0.09, 0.81 ± 0.02, 2.28 ± 0.25, 2.63 ± 0.55, and 0.52 ± 0.19, respectively (Figure 3B). Compared to the Nor group, the Con group showed significantly increased c-Fos protein expression (*p <* 0.01), while c-Fos protein levels in the EA, 650 LA, and 830 LA + EA group were significantly decreased compared to those in the Con group (*p <* 0.01).

The protein expression of CB1R in the Nor, Con, MA, EA, 650LA, 830LA, 650LA + EA, and 830LA + EA group was 0.98 ± 0.01, 3.98 ± 0.02, 4.64 ± 0.58, 3.87 ± 0.09, 2.88 ± 0.03, 6.46 ± 0.15, 6.76 ± 0.28, and 8.23 ± 0.37, respectively (Figure 3C). Levels were significantly higher in the Con (3.98 ± 0.02) than in the Nor group (0.98 ± 0.01, *p <* 0.01). Compared to the Con group (*p <* 0.01), levels were significantly lower in the 650LA group and significantly higher in the 830LA, 650LA + EA, and 830LA + EA group.

The protein expression of FAAH in the Nor, Con, MA, EA, 650LA, 830LA, 650LA + EA, and 830LA + EA group was 0.98 ± 0.01, 1.38 ± 0.51, 1.32 ± 0.09, 1.53 ± 0.19, 0.56 ± 0.01, 0.88 ± 0.01, 0.69 ± 0.02, and 0.24 ± 0.01, respectively (Figure 3D). Compared to the Nor group, the Con group showed a tendency to an increase in FAAH levels. The 830LA + EA group showed significantly decreased protein levels compared to the Con group (*p <* 0.05).

The protein expression of NeuN in the Nor, Con, MA, EA, 650LA, 830LA, 650LA + EA, and 830LA + EA group was 0.98 ± 0.01, 4.59 ± 0.83, 2.64 ± 0.60, 1.89 ± 0.43, 0.92 ± 0.01, 2.77 ± 0.49, 4.73 ± 0.98, and 0.55 ± 0.09, respectively (Figure 3E). Compared to the Nor group, the Con group showed significantly increased protein expression of NeuN (*p <* 0.01), while the EA, 650LA, and 830LA + EA group showed significantly decreased protein levels compared to the Con group (*p <* 0.05, *p <* 0.01, and *p <* 0.01, respectively).

The protein expression of synapsin I in the Nor, Con, MA, EA, 650LA, 830LA, 650LA + EA, and 830LA + EA group was 0.98 ± 0.01, 4.84 ± 0.31, 2.62 ± 0.15, 0.26 ± 0.06, 0.43 ± 0.06, 1.43 ± 0.14, 5.54 ± 1.48, and 0.15 ± 0.05, respectively (Figure 3F). Compared to the Nor group, the Con group showed significantly increased protein expression (*p <* 0.01). The MA, EA, 650LA, 830LA, and 830LA + EA group showed significantly decreased synapsin I levels compared to the Con group (*p <* 0.01).

The protein expression of MAP2 in the Nor, Con, MA, EA, 650LA, 830LA, 650LA + EA, and 830LA + EA group was 0.98 ± 0.01, 4.88 ± 0.47, 2.33 ± 0.31, 0.78 ± 0.15, 0.94 ± 0.07, 1.26 ± 0.07, 6.01 ± 0.79, and 0.11 ± 0.02, respectively (Figure 3G). Compared to the Nor group, the Con group showed significantly increased protein expression (*p <* 0.01), while levels were significantly lower in the MA, EA, 650LA, 830LA, and 830LA + EA group compared to the Con group (*p <* 0.01).

### Metabolome analysis

PCA was performed to observe metabolome changes according to treatment type in the allodynia model, and quality control samples pooled from all samples were concentrated near the center of the PCA score plot, indicating that the GC–MS metabolome analysis was reproducible and reliable. Our analysis of pattern changes after different types of acupuncture procedures at the two acupuncture points yielded unclear results.

**Figure 4:**
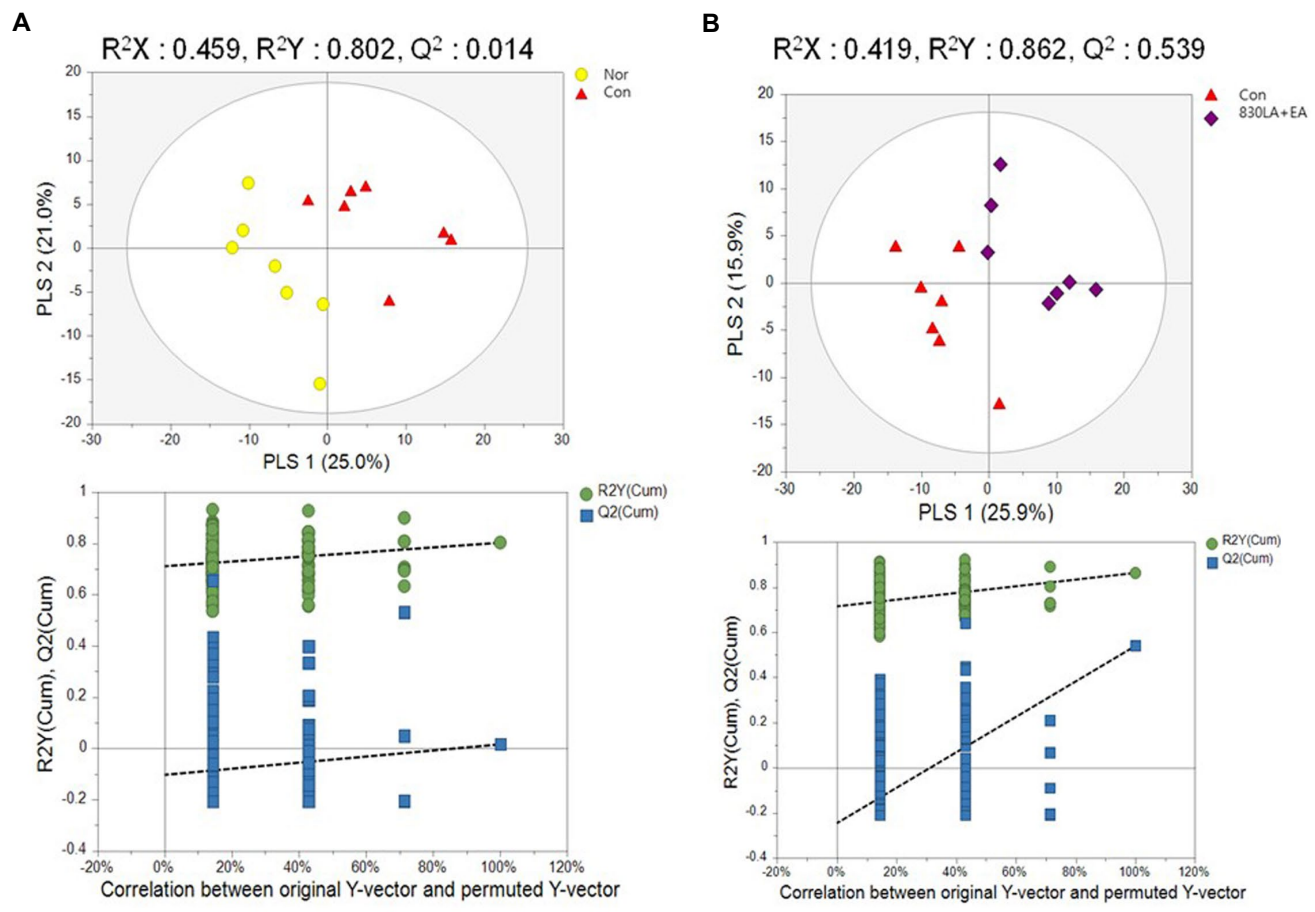
A PLS-DA score plot in rats with paclitaxel-induced allodynia according to type of acupuncture treatment. The Nor and the Con group show a tendency to separate, but the permutation test revealed no significant difference (*Q*^2^ = 0.0145, **A**). The PLS-DA result showed a pattern of separation between the Con group and the 830LA + EA group, and the permutation test showed a significant difference (*Q*^2^ = 0.5399, **B**). EA, electro acupuncture; LA, laser acupuncture; Nor, normal; Con, control.

PCA and PLS-DA were performed to assess metabolome changes between the Nor and the Con group and between the Con group and each treatment group. The PCA results showed a large deviation in the repeat interval without differences between the Nor and the Con group. The PLS-DA analysis showed a tendency to separate between groups, but the permutation test analysis showed no significant difference (*Q*^2^ = 0.0145; Figure 4A).

The PCA results yielded no difference between the Con group and each treatment group. The PLS-DA results showed a tendency to separate between groups, but the permutation test analysis revealed no significant difference except for the 830LA + EA group. Although PCA yielded no difference between the Con and the 830LA + EA group, the PLS-DA results showed a pattern of separation between the two groups, and the permutation test analysis indicated a significant difference (*Q*^2^ = 0.5399; Figure 4B).

## Discussion

This study shows significantly increased von Frey reaction times and force intensity in the MA, EA, and 650LA + EA group compared to the Con group on day 8. On day 15, the EA and the 830LA + EA group showed significantly increased von Frey reaction times and force intensity compared to the Con group. The von Frey filament quantifies the degree of pain, to ensure objectivity (31), and is used as an index to measure the degree of secondary hyperalgesia accompanying neuropathic or inflammatory pain (15). The von Frey index on day 8, when administration of paclitaxel was terminated, can be interpreted as indicating an analgesic effect, and the index on day 15, when the 9th treatment intervention was completed, seems to demonstrate an effect of damage repair and regeneration. The fact that the Con group had significantly decreased von Frey reaction times and force intensity on days 8 and 15 compared to the Nor group illustrates that paclitaxel blocks normal cell cycle progression and that apoptosis causes cell damage and allodynia. It seems that reaction times decreased because allodynia occurred and the animals felt pain, and that the force intensity decreased because movement was restricted by the pain. We assume that the MA, EA, and 650 LA + EA group exhibited a reliable analgesic effect, while the EA and the 830 LA + EA group demonstrated the effect of damage repair and regeneration. However, nociceptive and analgesic effects are affected by complex mechanisms including nociceptive, inflammatory, and neuropathic etiology and chemical and mechanical modality (32), it is important to comprehensively evaluate mechanical threshold values along with various factors related to pain in the body.

Among the three groups in which CB1R protein expression was significantly increased compared to the Con group, only the 650LA + EA group showed significantly increased mRNA expression of CB1R. CB1R is a cannabinoid receptor for a substance called anandamide, which is highly distributed in the central nervous system and involved in pain and neurodegeneration (7). It is mainly found in immune cells, such as B and T cells, and plays a role in immune responses and inflammation (33). Primarily expressed in presynaptic GABA and glutamate neurons, activation of CB1R by cannabinoids delivered from postsynaptic neurons can reduce the transmission of pain to the central nervous system by suppressing the release of GABA and glutamate in presynaptic neurons (34). In the current study, both mRNA and protein expression of CB1R were significantly increased when 650LA + EA treatment was applied, suggesting that the combination of 650-nm LA and EA can help reduce pain transmission. In addition, FAAH protein expression was significantly lower in the 830LA + EA group than in the control group. When endogenous cannabinoids bind to CB1R, they activate the receptor to induce analgesic action; otherwise, FAAH degrades the cannabinoids and the analgesic action does not last long (33). This therefore suggests a longer-lasting analgesic effect in the 830LA + EA group, which exhibited significantly lower FAAH protein levels than the other groups. The endocannabinoid system to which CB1R belongs is involved in the pain pathway and is composed of CB1R, CB2R, endocannabinoids such as transient receptor potential subfamily V member (TRPV) 1 and peroxisome proliferator-activated receptors (PPAR), and metabolizing enzymes (14). The endocannabinoid signaling system is regulated by synthesis and release of anandamide and 2-arachidonoylglycerol, uptake and degradation at supraspinal, spinal, and peripheral levels (8, 14, 35). Endocannabinoids are released activating CB1R, CB2R, and non-cannabinoid receptors including TRPV1 and PPAR (35). Released endocannabinoids are degraded by the hydrolyzing enzymes including FAAH and monoacylglycerol lipase, and by oxidative metabolism through cyclooxygenase, lipoxygenase and cytochrome P450 enzymes (8, 35). The endocannabinoid signaling mechanisms for modulating pain are related to enhancing endocannabinoids levels by inhibiting enzymes and blocking their reuptake, so further large-scale research is required to evaluate the complex therapeutic roles of the endocannabinoid system in various pain-related diseases.

This study also found significantly lower c-Fos protein levels in the EA, 650LA, and 830LA + EA group compared to the Con group. c-Fos is expressed in neurons in response to stimulation, and an increase in c-Fos is known to be associated with hypersensitivity to pain (36, 37). Compared to the Nor group, the Con group exhibited significantly increased c-Fos protein expression in the current study, which is consistent with a previous report that c-Fos protein is increased by noxious stimuli, such as thermal, mechanical, and chemical stimuli (9). These results indicate that treatment methods such as EA, 650LA, and 830LA + EA can have effects on pain relief in rats affected by allodynia, in line with the downregulation of c-Fos protein.

Only the 650LA + EA group showed a tendency to an increase in NeuN, synapsin I, and MAP2 levels compared to the Con group, although the difference was not significant. NeuN is a protein expressed in post-mitotic neurons that regulates neuronal growth (11, 12). Synapsin I is a type of neuronal phosphoprotein that binds to the cytoplasmic surface of synaptic vesicles and regulates synapse formation and neurotransmitter release through axonogenesis and synaptogenesis (10). MAP2 is a protein expressed by the MAP2 gene, and during cell division, microtubules extending from the centrosome bind to chromosomes to divide them (13). These three proteins are all involved in nerve regeneration, and the upregulation of the proteins in the 650LA + EA group therefore indicates the regeneration of paclitaxel-damaged neurons through therapeutic stimulation. 650LA + EA treatment can thus be expected to play an effective role in nerve regeneration.

The PLS-DA results of the metabolome analysis show a pattern of separation between the Con and the 830LA + EA group, and the permutation test analysis revealed a significant separation. These results indicate that treatment with 830LA + EA at the CV12 and ST36 acupoints brought about changes in the intestinal microflora and affected metabolomes. This suggests that 830-nm LA + EA could be more effective than other treatments in triggering changes in the microbial ecosystem in the large intestine through intestinal regulation.

This study has several limitations. First, the sample size for each group was relatively small. Second, among the six proteins used for our protein expression analysis, only mRNA for CB1R and FAAH could be confirmed. Additionally, correlation between proteins was not evaluated. Third, mRNA and protein expression was confirmed only in the spinal nerve tissues of rats, including the brain, spinal cord, and peripheral nerves. Lastly, pain assessment was performed using only the grimace scale. Despite these limitations, the present study shows the possibility of using LA combined with EA as an analgesic therapy for allodynia. We speculate that the combined treatment of EA and LA is helpful in pain relief and nerve regeneration, and induces changes in the intestinal microbiome.

In conclusion, the present study provides evidence that the combined treatment of EA and LA helps in pain relief and nerve regeneration and is effective in changing the intestinal microbiome. Among the different combination treatments, 650LA + EA induced upregulation of proteins related to pain relief and nerve regeneration, and 830LA + EA led to significant changes in metabolomes. These findings suggest the combined treatment of EA and LA as a useful method to relieve pain, such as in patients with allodynia. Further research is needed to assess the exact mechanism underlying the therapeutic effect of the combined treatment in pain-related diseases.

## Author’s note

This manuscript included a portion of a dissertation submitted by C-SY to the Department of Korean Medicine and the Graduate School of Dongshin University in partial fulfillment of the requirements for the degree of Doctor of Philosophy in Korean Medicine.

## Data availability statement

The original contributions presented in the study are included in the article/supplementary material, further inquiries can be directed to the corresponding authors.

## Ethics statement

The animal study was reviewed and approved by the Committee of Animal Care and Experiment of Dongshin University, Republic of Korea (DSU2020-04-01).

## Author contributions

C-SY, S-JK, and C-SN were involved in the study concept and design. M-HK carried out *in vivo* experiment. S-MK, C-KY, J-HY, E-JK, and H-SS performed the data analysis. C-SY, SP, and G-WL were involved in writing the manuscript. S-JK and C-SN were involved in critical revision for analyzing the results. All authors contributed to the article and approved the submitted version.

## Funding

This work is supported by the Korea Health Technology R&D Project through the Korea Health Industry Development Institute (KHIDI), funded by the Ministry of Health and Welfare, Republic of Korea (No. HI21C1924), the National Research Foundation of Korea (NRF), funded by the Ministry of Science and ICT (No. 2022M3A9B6017813).

## Conflict of interest

The authors declare that the research was conducted in the absence of any commercial or financial relationships that could be construed as a potential conflict of interest.

## Publisher’s note

All claims expressed in this article are solely those of the authors and do not necessarily represent those of their affiliated organizations, or those of the publisher, the editors and the reviewers. Any product that may be evaluated in this article, or claim that may be made by its manufacturer, is not guaranteed or endorsed by the publisher.
